# Tumor-Derived Exosomes in Immunosuppression and Immunotherapy

**DOI:** 10.1155/2020/6272498

**Published:** 2020-05-22

**Authors:** Wioletta Olejarz, Agnieszka Dominiak, Aleksandra Żołnierzak, Grażyna Kubiak-Tomaszewska, Tomasz Lorenc

**Affiliations:** ^1^Department of Biochemistry and Pharmacogenomics, Faculty of Pharmacy, Medical University of Warsaw, 02-097 Warsaw, Poland; ^2^Centre for Preclinical Research, Medical University of Warsaw, 02-097 Warsaw, Poland; ^3^1st Department of Clinical Radiology, Medical University of Warsaw, ul. Chałubińskiego 5, 02-004 Warsaw, Poland

## Abstract

Tumor-derived exosomes (TEX) are involved in cancer development, metastasis, and disease progression. They can modulate angiogenesis to elevate the malignant degree of tumor cells. TEX carry immunosuppressive factors affecting the antitumor activities of immune cells. Tumor cells as well as immune cells secrete immunologically active exosomes which affect intercellular communication, antigen presentation, activation of immune cells, and immune surveillance. Cell proliferation and immune response suppression create a favorable microenvironment for tumor. TEX can inhibit immune cell proliferation, induce apoptosis of activated CD8+ Teffs, suppress NK cell activity, interfere with monocyte differentiation, and promote Treg as well as MDSC expansion. Exosomes of microenvironment cells may also contribute to the development of drug resistance in cancer therapy. An important role of TEX in modulating the sensitivity of tumor cells to immunotherapy is a promising area of research to make the cancer therapy more successful.

## 1. Introduction

Exosomes are small extracellular vesicles (EVs) with 30–100 nm in diameter and the density of 1.10–1.14 g/ml [1]. Exosomes, as integral components of human blood, are secreted by many cell types, including immune cells and cancer cells. Exosomes have also been shown to be present in other body fluids [2], which creates the possibility of their potential use in diagnosis and therapy of diseases [3]. They are formed by a lipid bilayer membrane containing proteins, cholesterol, phosphatidylserine, ceramide, sphingolipids, and lipid rafts [4]. The proteins found in exosomes are involved in multivesicular body (MVB) formation (Alix, TSG101), membrane transport and fusion (annexins, flotillins, and GTPases), adhesion (integrins), and antigen presentation (MHC class I and II molecules). Moreover, tetraspanins (CD9, CD63, CD81, and CD82), heat shock proteins (HSP70, HSP90), and lipid-related proteins were found in exosomes. Exosomes contain short RNAs, long noncoding RNAs (lncRNA), viral RNAs, Y-RNAs, fragments of tRNAs, small nuclear RNAs, small nucleolar RNAs, and piwi-interacting RNAs [5, 6]. Intracellular endosome formation involves membrane surface proteins from the tetraspanin family, membrane signal molecules, endosomal-sorting complexes required for transport (ESCRT), and accessory proteins that assist in the final stages of exosome formation and secretion. Three ways of forming endosomes have been described: pathway depending on ESCRT and two ESCRT-independent pathways depending on tetraspanin and ceramid [7]. Exosomes internalize with target cells as a result of fusion, binding with surface proteins, or endocytosis [8]. The physiological state of the cell and the biogenesis pathway is responsible for the repertoire of particles packed in EVs [9, 10]. Tumor-derived exosomes (TEX) promote cancer progression via modification or suppression of the immune response and therapy resistance and may have immunotherapeutic applications [11]. TEX are involved in regulating peripheral tolerance in patients with cancer [12] and may serve as tumor biomarkers [13].

## 2. Composition of Cancer Exosomes

TEX are involved in cancer development, tumor progression, promoting angiogenesis, and migration of tumor cells during metastasis and thus are recognized as multifaceted regulators of cancer development [14, 15]. They are considered being carriers of molecules determining the formation of premetastatic niche in the target organ that leads to the right metastasis of primary metastatic cells [16]. Cancer EVs could change the phenotype of normal, noncancerous cells [17] or trigger a transient transformation [18], or they can increase the genotoxic stress thereby provoking genetic instability or transfer individual oncogenes [19]. Exosomal integrins determine the metastatic sites of the primary tumor cells, mediate the interaction of exosomes and specific resident cells of the targeted organ, regulate the function of targeted cells by activating protooncogenic proteins, and may be essential for tumor progression [20]. Exosomes released from stromal cells have been shown to be able to stimulate nearby tumor cells to metastasize. They also promote tumor cell proliferation and inhibit their apoptosis [21]. It was shown that type II transmembrane protein, Fas ligand (FasL), present in the structure of exosomes released from cancer cells, stimulates T cell apoptosis and is cytotoxic to natural killer (NK) cells [22].

### 2.1. RNA Content of Cancer Exosomes

Long noncoding RNA is one of the types of RNA present in the structure of exosomes [23, 24]. This type of RNA does not encode any proteins but participate in chromosome modification, gene transcription, mRNA translation, and the regulation of protein biological function [25]. Exosomal lncRNAs play critical roles in facilitating tumorigenesis by regulating angiogenesis, immunity, and metastasis [26]. Studies carried out on hepatic cancer stem cells have shown that exosomes released from them contain lncRNAs enhancing expression of vascular endothelial growth factor receptor 1 in endothelial cells, which promotes angiogenesis [27]. Ni et al. demonstrated that breast cancer-derived exosomes transmit lncRNA SNHG16 to induce CD73+*γδ*1 Treg cells which have immunosuppressive functions [28]. Also, Liang et al. have shown that lncRNA RPPH1 promotes colorectal cancer metastasis by interacting with TUBB3 and by promoting exosomes-mediated macrophage M2 polarization [29].

TEX containing microRNA (miRNA) seem to play a special role in the development and expansion of tumors. Scientists found that this type of EVs mediates the stimulation of cancer in noncancerous epithelial cells and then induces tumor metastasis. In the case of breast cancer, a relationship between tumor invasion and miRNA-rich exosomes secreted from tumor-associated macrophages has been demonstrated. Exosomes isolated from patients' blood and breast cancer cell lines have been reported to carry miRNAs that induced the transformation and formation of tumors in noncancerous breast cells [30]. Zhou et al. demonstrated that in patients with this type of cancer, exosomal miR-105 promotes metastasis by destroying endothelial cell barriers [31]. Fong et al. showed that the TEX containing miRNA-122 significantly reduce glucose uptake by noncancer cells. It is a manner of providing access to the basic energy substrate for cancer cells [32].

miRNAs, including those traveling inside exosomes or those released by tumor cells into the circulation, may serve as promising biomarkers in colorectal cancer [33]. It was shown that serum exosomal miR-320d is a promising noninvasive diagnostic biomarker for distinguishing metastatic from nonmetastatic colorectal cancer [34]. miRNA-21 and lncRNA SNHG1 (small nucleolar RNA host gene 1) may serve as potential diagnostic, prognostic biomarkers and therapeutic targets for esophageal squamous cell carcinoma patients [35]. Exosomal microRNAs have a key role in the tumor microenvironment of breast cancer and may serve as molecular markers [36]. Their analytical stability constitutes exosomal miRNAs as promising molecular markers to overcome numerous limitations of cancer clinical management [37].

The clinical value of exosomes contained in saliva and urine has also been demonstrated in the diagnosis of pancreatic and biliary tract cancer and prostate cancer, respectively [21]. Circular RNA (circRNA) was also found in the structure of TEX. This type of RNA acts as miRNA sponge and now is recognized as a key factor in tumor development. Zhang et al. have found that exosomal circRNA secreted from adipocytes promotes the growth of hepatocellular carcinoma [38].

### 2.2. DNA Content of Cancer Exosomes

In recent years, there have been reports indicating the presence of DNA in exosomes (exoDNA) [39–41]. It has been suggested that exosomal DNA may play an important role in genetic communication between different cells. The mechanism of DNA packaging to exosomes is not yet fully understood. Experiments conducted by Guescini et al.'s team in 2010 showed for the first time the presence of mitochondrial DNA (mtDNA) in exosomes secreted by astrocytes and glioblastoma cells [42]. It is worth noting that Kawamura et al. showed that a significant proportion of the detected mtDNA occurs on the surface of exosomes, but the significance of this location is unknown [43]. It has been suggested that this type of DNA may stimulate exosomal aggregation and possibly affect their function in recipient cells [44]. In turn, studies of exosomes released from glioblastoma and medulloblastoma cells under culture and *in vivo* conditions showed the presence of single-stranded DNA (ssDNA), referred to as exoDNA, including both genomic DNA (gDNA), complementary DNA, and transposonal DNA, probably derived from cytoplasmic and nuclear compartments. The source of the mentioned ssDNAs is indicated: amplified cytogenetically appearing sequences as so-called small “double-minute” chromosomes, abnormal DNA replication in cancer cells, and reverse transcription of cellular RNA.

In 2014, Thakur et al.'s team published data indicating that most of the DNA present in exosomes derived from cancer cells (melanoma, breast, lung, prostate, and pancreatic cancer) is double-stranded [45]. Interestingly, mitochondrial DNA has not been reported in these cells. It was also shown that the amount of exoDNA in normal human dermal and mammary fibroblasts was significantly lower than the amount of exoDNA isolated from tumor cells [45]. Exosomal DNA analysis allows the detection of tumor-specific mutations, which, in the case of heterogeneous solid tumors, can give a more accurate picture of tumor genetics overall than small tissue biopsies [46].

The presence of the mutated epidermal growth factor receptor (EGFR) alleles in exoDNA, especially mutations such as *exon 19 Del* and *exon 21 L858R*, characteristic of lung cancer cells, may be important when selecting patients for targeted therapy using a tyrosine kinase inhibitor [40, 47]. It has also been shown that mtDNA present in fibroblast-derived exosomes activates oxidative phosphorylation (OXPHOS) in breast cancer cells, which leads to resistance to hormone therapy in OXPHOS-dependent breast cancer [48]. Thus, exosomal DNA can be not only a marker for early detection of cancer but also a parameter enabling treatment selection and monitoring.

The possibility of a relationship between the presence of gDNA in exosomes and processes such as cell aging and stimulation of the inflammatory pathway cGAS/STING is also suggested [49]. Kitai et al. have shown that exosomal DNA can activate innate antiviral response of immune cells, while Lian et al. demonstrated that exosomal DNA mediates activation of the AIM-2 inflammasome pathway in dendritic cells (DC) and the production of interleukins 1*β* and 18 in patients with enteritis treated with irinotecan [50, 51].

The above issues require further research, because in one of the latest publications from 2019, Pluchino and Smith question the presence of DNA in exosomes [52]. The authors suggest that DNA referred to as exosomal is in fact a nucleic acid-histone complex released by autophagy, subjected only to coisolation with exosomes [52].

### 2.3. Protein Content of Cancer Exosomes

According to the Exosomal Database (http://www.exocarta.org), 9769 proteins were identified in exosomes isolated from many different cell types of many organisms. These are both constitutive components of exosomes (e.g., tetraspanin and the ESCRT complex), membrane transport and fusion proteins, adhesive proteins, heat shock proteins, enzymes, and receptor proteins, as well as major histocompatibility complex (MHC) class I and class II proteins, integrins, programmed-cell death ligand 1 (PD-L1), EGFR, tumor-necrosis-factor-related apoptosis-inducing ligand (TRAIL) specific for the type of cell that secretes these vesicles, etc. [53].

In gastric cancer, the effect of such exosomal proteins as tetraspanin-8 and DC97 on the growth and metastasis of cancer cells has been proven. In addition, tetraspanin-8 can be used as an early marker for the development of this cancer [54]. In hepatocarcinoma (HCC), it has been shown that proteins present in exosomes can play a role in cell migration (receptor tyrosine kinase-MET, caveolin 1 and 2), cell invasion (MET, S100 calcium binding protein A4), and angiogenesis (S100 calcium-binding protein A11) [55]. A similar effect in melanoma has interleukin-6 (IL-6), vascular endothelial factor (VEGF), and matrix metalloproteinases (MMP) transported by exosomes [56]. Immunosuppressive effects of melanoma cell-derived exosomes on primary human immune cells were reported in melanoma patients [57]. It was shown that melanoma cell-derived exosomes are enriched in immunosuppressive proteins, inhibit CD69 expression, induce apoptosis, and suppress proliferation in CD8+ T cells and downregulates NKG2D expression in NK cells. Melanoma cell-derived exosomes emerge as the major mechanism of tumor-induced immune suppression and as an underestimated barrier to successful melanoma immunotherapy [57]. Studies in murine breast tumor lines of a different tendency to metastasize have shown significant differences in the proteomic composition between the exosomes secreted by metastatic and nonmetastatic cells. In the structure of exosomes derived from metastatic cells, there were primarily proteins involved in the processes of proliferation, migration, and angiogenesis, including membrane proteins, ceruloplasmin and metadherin, directing primary cancer cells to specific metastatic sites. Exosomes secreted by nonmetastatic cells mainly contained proteins involved in cell adhesion [58].

Proteins contained in exosomes may participate in the promotion and metastasis of tumors but may also be used as early markers of these diseases [54]. Clark et al. [59], Jakobsen et al. [60], and Li et al. [61] reported exosomal CD317, EGFR, and human leucine-rich alpha-2-glycoprotein 1 (LRG1) as potential biomarkers of non-small-cell lung cancer. It is also suggested to use protein 1 associated with adenyl cyclase (CAP1) contained in exosomes derived from HCC cells as a marker associated with HCC metastasis and recurrence [62]. Higher expression of prostate cancer (PC) markers such as prostate-specific antigen (PSA) and survivin in exosomes has also been demonstrated compared to blood serum. Thus, exosomal PSA may become a more reliable marker of this cancer. In addition, survivin present in exosomes enables the differentiation of PC and prostatic hyperplasia [63].

Analysis of the protein profile of TEX may also play a role in the selection and monitoring of therapies [64]. Exosomes as particles with potential effects on the antitumor immune response may participate in anticancer therapy as carriers of antigens in the immunotherapy of cancer [65]. Tucci et al. showed a correlation between high levels of CD28 and programmed death receptor 1 (PD-1) and the therapeutic response to anti-CTLA4 immunotherapy in metastatic melanoma [56].

## 3. TEX in Immunosuppression

**Figure 1:**
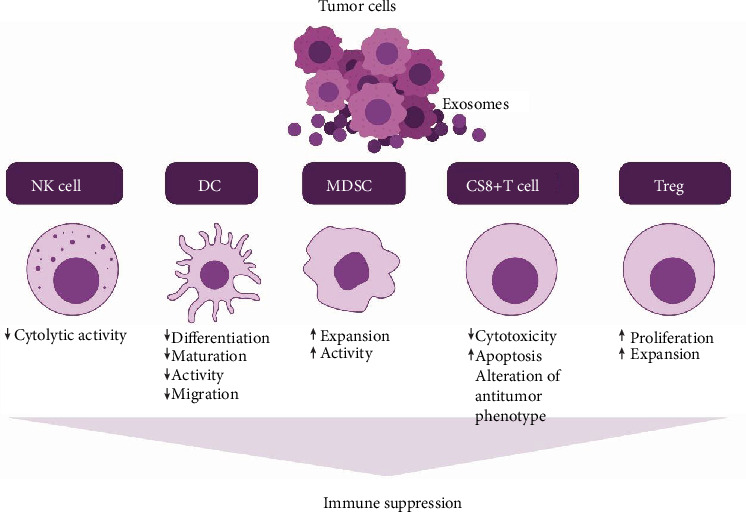
TEX suppress NK cell activity, promote MDSC expansion, inhibit immune cell proliferation, induce apoptosis of activated CD8+ T cells, and promote Treg expansion.

TEX interacting with immune cells deliver negative signals to these cells and interfere with their antitumor functions. By suppressing functions of immune effector cells, TEX promote tumor progression and facilitate tumor escape [66]. They carry immunosuppressive molecules and factors which directly or indirectly influence the development, maturation, and antitumor activities of immune cells [67] (Figure 1).

### 3.1. TEX Inhibit Immune Cell Proliferation and Induce Apoptosis of Activated CD8+ Teffs

TEX modulate the activity of T cells through inhibiting signaling and proliferation as well as promoting apoptosis of CD8+ T cells [68]. On the other hand, exosomes derived from immune cells promote proliferation of all T cells [69]. Furthermore, studies have demonstrated that tumor can change the antitumor phenotype of cytotoxic CD8+ T cells towards potent suppressor function of the mentioned cells [70, 71]. A new phenotype is associated with the loss of CD27/28 expression and can be induced by TEX. These novel suppressive abilities were shown not only in tumor T cell *in vitro* coculture assays but also in CD8+ T cells obtained from the tumors of patients with head and neck cancer [72]. Moreover, exosome-derived RNA transferred into normal CD8+ T cells can induce the phenotype alterations.

Tumor cells are characterized by FasL expression. Transmembrane protein FasL is involved in apoptosis promotion, downregulation of immune response, and control of self-antigen tolerance through the interaction with receptor Fas [73]. It is reported that TEX with FasL expression are able to cause antitumor CD8+ T cell apoptosis [74, 75]. This ability to induce apoptosis of cytotoxic T cells can also be due to the expression of MHC class I molecules in TEX. It is suggested that the interaction of MHC class I with the CD8 receptor leads to the apoptosis of T cells via the activation of the Fas/FasL signaling pathway [76]. Chen et al. have reported that abundant exosomes released from metastatic melanoma express PD-L1 which is upregulated by IFN*γ* [77]. Cordonnier et al. confirmed that PD-L1 levels in circulating exosomes seem to be a more reliable marker than PD-L1 expression in tumor biopsies. Circulating exosomal PD-L1 may be useful to monitor a melanoma patient and predict the tumor response to treatment [78]. Also, Del Re et al. demonstrated that PD-L1 mRNA expression in plasma-derived exosomes was associated with response to anti-PD-1 antibodies in melanoma and non-small-cell lung carcinoma (NSCLC) [79]. PD-L1—apoptosis-inducing molecule—interacts with PD-1 expressed on CD8+ T cells leading to the suppression of these cells' function and causing the tumor growth. The correlation of PD-L1 levels on TEX with disease activity was demonstrated in patients with head and neck cancer [80].

### 3.2. TEX Suppress NK Cell Activity

Natural killer cells and natural killer T (NKT) cells are subsets of lymphocytes and can respond to the presence of tumor cells and participate in antitumor immune responses [81]. Invariant natural killer T (iNKT) cells are a unique innate T lymphocyte population which plays an important role in the immune surveillance of blood cancers [82]. NK cell dysfunctions have been observed in various hematologic malignancies, including chronic lymphocytic leukemia (CLL) through soluble ligand BAG6/BAT3. It was shown that exosomal vs. soluble BAG6 expression may cause immune evasion of CLL cells [83]. Activating receptors such as NKG2D, NKP30, NKP46, and NKG2C play an important role in NK cell cytotoxicity [84]. It was shown that expression of NKG2D was reduced markedly in large numbers of tumor-infiltrating and peripheral blood T cells from individuals with cancer [85]. NKG2D is also an activating receptor for NKT, CD8(+), and gammadelta(+) T cells. It was shown that exosomes derived from cancer cells express ligands for NKG2D resulting in downregulation of NKG2D on these cells and impaired cell cytotoxic function [86]. This effect might be caused by TGF-*β*1 secretion by tumor-derived exosomes [87].

### 3.3. TEX Promote Treg Expansion

Regulatory T cells (Tregs) play a crucial role in the suppression of immune system. Tumor-infiltrating Tregs as well as their elevated level in peripheral blood of patients indicate strong prognostic significance in a cancer [88, 89]. Increased levels of Tregs and TEX were found in the serum of myeloma patients [90, 91]. *In vitro* studies have shown that TEX induced immune suppression by promoting expansion of CD4(+)CD25(+)FOXP3(+) T regulatory cells and the demise of antitumor CD8(+) effector T cells [68]. TGF-*β*1 enhances the conversion of CD4+CD25- T cells to CD4+CD25+FOXP3+ Tregs, and TEX participate in this process as the application of neutralizing antibodies against TGF-*β*1 or IL-10 results in the inhibition of Treg expansion. Coincubation of Tregs with TEX leads to a higher expression of IL-10, TGF-*β*1, FasL, and CTLA-4 on Tregs, and TEX augment Treg resistant to apoptosis [91].

### 3.4. TEX Promote MDSC Expansion

Myeloid-derived suppressor cells (MDSCs) are immature suppressive cells with an ability to promote tumor progression in cancers [92]. They influence the tumor microenvironment by depletion of amino acids, oxidative stress, decreased trafficking of antitumor effector cells, and increased regulatory T and regulatory dendritic cell responses [93]. The peripheral blood MDSC level correlates with clinical stage and therapeutic response in various chemoresistant cancers [94]. TEX have been revealed to enhance the development of MDSCs, among others, facilitating their activation, promoting the expansion, and enhancing the immunosuppressive function [95]. Accumulation of MDSCs could negatively affect the antigen processing and presentation and produce numerous immunosuppressive inhibitory factors which cause T cell apoptosis [96]. It was shown that glioma cells influence the differentiation and activation of MDSCs via exosomes by hypoxia-inducible expression of miR-10a and miR-21 [97]. Gastric cancer-secreted exosomes are able to deliver miR-107 to the host MDSCs where they induce their expansion and activation by targeting DICER1 and PTEN genes [98].

### 3.5. TEX Interfere with Monocyte Differentiation

TEX suppress the immune system through affecting monocyte maturation and differentiation [99]. The key role of dendritic cells is capturing antigens, subsequent maturation, and finally migration to lymph nodes where antigen presentation and T cell stimulation take place. However, the tumor microenvironment is characterized by suppressed dendritic cells and inhibited their immune response [100]. TEX block dendritic cell migration to secondary lymphoid organs through the inhibition of chemokine receptor expression [101]. Valenti et al. have reported that exosomes released by human colorectal carcinoma and melanoma cells inhibit the differentiation of human monocyte precursors to dendritic cells [102]. Colorectal cancer-derived microvesicles may modulate differentiation of human monocytes to macrophages [103]. Monocytes after membrane fusion with TEX gain a new phenotype without human leukocyte antigen-DR (HLA-DR) expression and costimulatory molecule upregulation but with CD14 expression, called CD14+HLA-DR^lo/neg^ monocytes and have emerged as important mediators of tumor-induced immunosuppression [104]. TEX cause immune suppression in monocytes through altered STAT3 signaling and induction of arginase expression and reactive oxygen species [105]. TEX may trigger an antitumor response by transferring tumor antigens to immune cells such as monocytes, macrophages, and dendritic cells. It was shown that the differentiation status of these cells influences the efficiency of EV uptake [106].

## 4. TEX in Cancer Immunotherapies

TEX have the ability to suppress proliferation and differentiation of immune cells, including even changing their function and remodeling the genetic material. They carry a variety of tumor antigens or invasive-related molecules which impede effective response to cancer immunotherapy [107]. Tumor-associated antigens (TAA) are an example of the TEX cargo which lead to a decrease in effectiveness drug therapy of cancer. TAA bind antibodies (Abs) produced against cancer cells effectively, and therefore, small and insufficient amounts of antibodies reach the cancer tissue. Trastuzumab is frequently used in breast cancer therapy, and it is proved that exosomal nanoparticles are interfering with the therapeutic activity of the trastuzumab in this model of treatment [108]. Furthermore, TEX are able to suppress Ab-dependent cell-mediated cytotoxicity (ADCC). ADCC has a prominent role in cancer prevention, and it is one of the main mechanisms of therapeutic antitumor activity of the humanized antibody [109].

During cancer progression, CD8 cytotoxic T lymphocytes (CTL) encounter dysfunction and exhaustion due to immune-related tolerance and immunosuppression within the tumor microenvironment (TME) and favor adaptive immune resistance. PD-L1 and CTL-associated antigen 4 (CTLA-4) are checkpoint receptors that can be targeted for relieving exhaustion of CD8 T cells and thereby eliminating cancer cells [110]. PD-L1 on the surface of tumor cells binds its PD-1 receptor on effector T cells, thereby suppressing their activity. Antibody blockade of PD-L1 can activate an antitumor immune response leading to durable remissions in a subset of cancer patients. These findings show that exosomal PD-L1 represents a new therapeutic target, which could overcome resistance to current antibody approaches [111]. Monoclonal antibodies are used to stimulate the function of the immune system in antitumor immunotherapy. Checkpoint inhibitors are an example of this treatment strategy through blocking the interaction between PD-1 and PD-L1. The protein blockade in question encourages proper functioning of CD8 T cells and their effective fight against cancer, and as it turned out, exosomes containing PD-L1 may be predictors for anti-PD-1 therapy [77]. PD-1/PD-L1 blockade to c-Met-overexpressing cancer cells is a promising strategy for the treatment of gastric cancer and potentially other malignancies [112]. It was shown that T cell immunoglobulin- and mucin-domain-containing molecule 3 (Tim-3) is the next-generation immune checkpoint that can be activated by its ligand Galectin-9 to negatively regulate the antitumor immune response. Exosomal proteins, especially exosomal TIM-3 and Galectin-9, could be potential biomarkers for non-small-cell lung cancer [113].

Exosomal PD-L1 has an important function in tumor metastasis, immune escape, and immunotherapy, but some issues remain to be clarified. Main complications linked to PD-L1 evaluation is adjusting an appropriate test and obtaining an appropriate number of tumor cells in specimens [114]. The Blueprint PD-L1 IHC Assay Comparison Project revealed difficulties in the interpretation of IHC detection of PD-L1 and concordance of three assays and indicated the necessity for further studies [115]. Patients treated with PD-1/PD-L1 blockade have significant individual differences. Exosomal PD-L1 which is resistant to immunotherapy may be due to the low abundance relative to surface PD-L1. Additionally, we know nothing about whether the function of exosomal PD-L1 is cancer-type dependent or not [116]. The controversies concerning the examination of PD-L1 expression on cancer cells as a predictive factor are numerous and include technical, diagnostic, and methodical issue [117, 118]. Further clarification of the role of exosomal PD-L1 in tumor progression may contribute to the early diagnosis and treatment of cancer.

## 5. Exosomes Deriving from Immune Cells as Stimulators of Cancer Therapy

The immunological activities of exosomes affect immunoregulation mechanisms including modulating antigen presentation, immune activation, immune surveillance, intercellular communication, and immune suppression [119]. CD8+ T cells, once receiving tumor-specific antigenic stimulation by dendritic cells, are activated and differentiated into effector CTL. It was shown that DC-derived exosomes promote but Treg-derived exosomes inhibit CTL generation [120]. Moreover, NK cells show strong cytotoxicity against tumor cells. As it turned out, FasL expressed on the membrane of NK cell-released exosomes may play a part in killing of Fas+ tumor cells [121].

Research has shown that exosomes participate in adoptive cell therapy (ACT). It is a type of genetic immunotherapy based on redelivering tumor-infiltrating lymphocytes (TIL) as anticarcinogenic immune cells following their *ex vivo* multiplying. Exosomes deriving from immune cells render the method effective by their antitumor properties as well as exerting a cytotoxic effect on cancer cells [122]. With this idea, Chinese scientists have developed a new class of cancer vaccines—cell-free tumor vaccines contain *α*-fetoprotein-enriched DC-derived exosomes [123]. They stimulate cells of the immune system to produce IFN-*γ* and IL-2 and reduce the expression of TGF-*β* and IL-10 at the tumor site. This leads to the induction of the antigen-specific response to cancer cells and thus inhibition of tumor growth and limitation of its metastatic ability. Attempts to use exosome's antineoplastic properties involve also exosomes released by natural killer cells [124]. It has been proved that that mentioned nanovesicles are miR-186 transporters that downregulate MYCN and TGFBR1 expression in neuroblastoma. This, in turn, suppresses spreading and TGF*β*-dependent immune escape mechanisms in neuroblastoma. Furthermore, exosomal heat shock protein (HSP70) promotes NK cell activation [125]. This fact enforces the theory about immunostimulatory exosomes in cancer therapy.

Bioengineered exosome designing is a new area of research based on inserting antitumor antigens in the cell membrane of TEX which contribute to antigen-specific responses against tumor cells [126]. Further modifications of tumor-derived nanovesicles concern ability to work as new drug carriers, especially biological therapeutics, which are unable to penetrate the cell membrane because of its sensibility to degradation or destruction of biological therapeutics by the immune system [127]. Designing immunostimulatory exosomes or impairing the suppressive effect of TEX molecules at least is a promising avenue for the exosome-based immunotherapeutic approaches to cure cancer.

## 6. TEX and Drug Resistance in Cancer Therapy

Exosomes can promote therapy resistance in cancer cells through cell-cell communication within the tumor microenvironment [128]. For example, macrophage exosomes initiate drug resistance in epithelial ovarian cancer therapy occurring under hypoxic conditions of the tumor microenvironment. The results of studies by Ciravolo et al. showed that exosomes secreted by HER2-overexpressing breast cancer cell lines are bound to trastuzumab which inhibits their anticancer cell proliferative activity [108]. It was also reported that exosomes secreted from M2 macrophages containing miRNA-21 determined cisplatin resistance in gastric cancer cells. Conversely, chemotherapy can promote tumor metastasis. An Italian group described a set of microRNAs (miR-146a, miR-155, miR-125b, miR-100, let-7e, miR-125a, miR-146b, and miR-99b) that are associated with myeloid-derived suppressor cells and resistance to treatment with immune checkpoint inhibitors in melanoma patients [129]. The miRs are mediated by melanoma-derived exosomes and are responsible for the conversion of monocytes into MDSCs and recreativeness of MDSC features upon transfection [129]. The studies on an animal model of breast cancer have shown that exosomes of cancer cells released under the influence of chemotherapy promote the formation of premetastatic niche in the lungs [20, 27, 130, 131]. TEX play a key role in protumor immunity and disease progression [132]. They carry immunosuppressive molecules and factors affecting the development, maturation, and antitumor activities of immune cells [11]. TEX demonstrate a dual effect of cancer response to immunotherapy by transport invasive-related or metastasis molecules; on the other, they have the ability to elicit tumor antigen-specific immune response and cause tumor cell lysis [133]. An important role in modulating the sensitivity of tumor cells to immunotherapy is a promising area of research to make the cancer immunotherapy more successful [134].

## 7. Conclusions

TEX are key mediators in intercellular communication which play a crucial role in the tumor microenvironment. They carry immunosuppressive cargos, deliver molecular signals to immune cells, and are involved in varieties of immunosuppressive or immune-stimulative signaling pathways. TEX may interfere with immune therapies either by sequestration of therapeutic antibodies or elimination of vaccine-induced or adoptively transferred immune effector cells. Understanding of exosome's immunosuppressive or activating influence on cancer immunotherapy may contribute to generating effective methods of therapies and give an answer to the question why several tumors do not respond to today's treatment options. It is considered that TEX's immunosuppressive or activating effect depends on their contents, immune cell condition, and tumor's environment where the extracellular vesicles are created by stem cells. Importantly, immune escape of tumors has been considered a major barrier to successful immunotherapy of cancer.
